# Lectin Staining of Microvascular Glycocalyx in Microfluidic Cancer Cell Extravasation Assays

**DOI:** 10.3390/life11030179

**Published:** 2021-02-25

**Authors:** Sebastian Beyer, Anna Blocki, Matthew Chung Yin Cheung, Zoe Ho Ying Wan, Babak Mehrjou, Roger Dale Kamm

**Affiliations:** 1 Department of Biomedical Engineering, The Chinese University of Hong Kong, New Territories, Hong Kong, China; 1155126643@link.cuhk.edu.hk (M.C.Y.C.); babakmehrjou@cuhk.edu.hk (B.M.); 2 Institute for Tissue Engineering and Regenerative Medicine, The Chinese University of Hong Kong, New Territories, Hong Kong, China; anna.blocki@cuhk.edu.hk (A.B.); wanhoying@link.cuhk.edu.hk (Z.H.Y.W.); 3 Faculty of Medicine, School of Biomedical Sciences, The Chinese University of Hong Kong, New Territories, Hong Kong, China; 4 Department of Orthopaedics and Traumatology, Faculty of Medicine, The Chinese University of Hong Kong, Shatin, Hong Kong, China; 5 Departments of Biological Engineering and Mechanical Engineering, Massachusetts Institute of Technology, 500 Technology Square, MIT Building, Room NE47-321, Cambridge, MA 02139, USA

**Keywords:** endothelial glycocalyx, cancer cell extravasation, lectin staining

## Abstract

The endothelial glycocalyx forms the inner-most lining of human microvasculature. It ensures the physiological function of blood vessels and plays a crucial role in the occurrence and progression of microvascular diseases. The present communication aims to highlight the usefulness of high-resolution imaging of lectin (*Bandeiraea Simplicifolia*) stained endothelial glycocalyx in 3-dimensional microfluidic cell cultures. The microfluidic system allowed visualizing cancer cell extravasation, which is a key event in metastasis formation in cancer pathologies. In brief, microvascular networks were created through spontaneous vasculogenesis. This occurred from 3 dimensional (3D) suspensions of human umbilical vein endothelial cells (HUVECs) in hydrogels confined within microfluidic devices. Extravasation of MDA-MB-231 breast cancer cells from perfusable endothelial lumens was observed with confocal imaging of lectin-stained microvascular networks. The present work provides guidance towards optimizing the methodology used to elucidate the role of the endothelial glycocalyx during cancer cell extravasation. In particular, a high-resolution view of the endothelial glycocalyx at the site of extravasation is presented. The occurrence of glycocalyx defects is well aligned with the contemporary notion in the field that glycocalyx shedding precedes cancer cell extravasation.

## 1. Introduction

Providing new methodologies such as microfluidic cell culture is instrumental to better understand the role of the endothelial glycocalyx in complex biological processes such as cancer cell extravasation.

The endothelial glycocalyx is a brush-like structure comprising glycoproteins on the inner lining of microvasculature [1]. Core proteins anchor negatively charged and hydrated carbohydrate chains to endothelial cells. The carbohydrate chains protrude into the vascular lumen and comprise hyaluronic acid (HA), heparan sulfate (HS), and chondroitin sulfate (CS) [2]. These carbohydrate chains are decorated with sialic acid (SA) at their terminal ends [2,3]. A healthy, undegraded endothelial glycocalyx forms a repulsive barrier between the liquid tissue within the vessels and the solid tissue in the perivascular space. The glycocalyx also regulates the hemorheological properties of blood vessels [4]. The endothelial glycocalyx actively contributes towards the selected transport of cells, proteins, and nutrients across the endothelial barrier to ensure tissue homeostasis.

The migration of blood-borne cells such as neutrophils or macrophages across the endothelial barrier is a regulated process. Extravasation of these cells is receptor-mediated and inhibited through the glycocalyx by shielding the responsible receptor molecules on the endothelial surface [5]. During physiological events such as tissue inflammation, endothelial cells shed their glycocalyx via a cytokine-mediated process. This allows neutrophils and macrophages to infiltrate the inflamed tissue [6].

Cancer cell extravasation, on the other hand, follows a different, less intuitive course of action. Cancer cells dislodge from the primary tumor, entering the vascular system to become circulating tumor cells (CTCs). These CTCs often extravasate across seemingly healthy endothelial barriers into healthy tissue to form metastasis distant from the primary tumor [7]. The process of cancer cell extravasation has been the subject of detailed investigation and extravasation is often observed in the microvasculature. The small diameter of microvessels causes cancer cells to get entrapped mechanically prior to any possible interaction with the glycocalyx or other components of the endothelial barrier [8].

Investigations on the cancer-endothelial cell interface in two-dimensional cell cultures have shown damaged and dysfunctional glycocalyx in the presence of cancer cells [2,6]. It was shown that human malignant breast cancer cells (MDA-MB-231) had stronger adhesion to the endothelium upon enzymatic degradation of HS [2,7]. Similar observations were made for human lung carcinoma cells (A549) upon removal of HA [2,8]. Furthermore, it was shown that excessive SA-degrading enzyme, neuraminidase (Neur) sheds the α-2,6-linked, α-2,3-linked, and α-2,8-linked SA residues and degrades the whole endothelial glycocalyx leading to an increase in metastatic cancer cell attachment to the endothelium [3]. Indeed, the consensus in the field appears to be that endothelial glycocalyx shedding precedes cancer cell extravasation. However, the detailed and accurate spatiotemporal role of the endothelial glycocalyx on cancer cell extravasation is not yet fully understood. One of the reasons for this limited spatiotemporal understanding of endothelial glycocalyx dynamics during cancer cell extravasation is the difficulty of observing this process in non-physiological 2D cell cultures. Tissue sections allow the study of a snap-shot of the endothelial glycocalyx using rapid freezing and freeze substitution transmission electron microscopy methodologies developed by the groups of John Tarbell and Eno E. Ebong [9]. Animal models allow the study of cancer cell invasion in complex tissues employing intravital microscopy techniques as for example reported by Peter Friedl and co-workers [10]. However, the timing of a single extravasation event, as well as manipulation of the endothelial glycocalyx, is difficult in vivo.

Microfluidic cell culture assays, on the other hand, allows for dissecting complex biological processes. Microfluidic interfaces in multicellular culture allow detailed study of cellular interaction [11]. Emulating human microvasculature within microfluidics requires growing microvascular networks (MVNs) with luminous structures typically having less than 100 µm in diameter. MVNs with these dimensions can be generated from 3 dimensionally suspended endothelial cells in hydrogels through spontaneous vasculogenesis [12]. Functional and perfusable MVNs with openings to both side channels of the microfluidic device were previously created using human umbilical vein endothelial cells (HUVEC) with direct co-cultures of human mesenchymal stem cells (hMSCs) or spatially separated fibroblast culture [11,13,14]. However, perfusable MVNs within the hydrogel portion may also be obtained in pure endothelial cell culture. This is envisioned to be beneficial in studying certain biological interactions that are sensitive to perturbation by paracrine signaling.

In this study, preliminary observations on the role of the endothelial glycocalyx during cancer cell extravasation in 3D MVNs in microfluidic devices are presented. The focus of the present study is the evaluation of the general suitability of lectin staining for high-resolution imaging in microfluidics. This may pave the way for future, more quantitative studies with spatiotemporal resolution.

## 2. Materials and Methods

### 2.1. Microfluidic Device Design, Fabrication, and Cell Culture

The microfluidic design was chosen to have a central gel region that is confined by trapezoidal posts and framed by channels for media exchange. The height of the central gel region was 120 µm and the design was adapted from earlier work [15]. Microfluidic devices were coated with poly-D-lysine, a polyelectrolyte to facilitate protein absorption through electrostatic forces similar to the layer-by-layer polyelectrolyte multilayer build-up described by some of us [16]. Microfluidic device fabrication followed previously published protocols [17,18]. Here, protocols were adapted by using high-intensity UV treatment for 20 min for sterilization immediately prior to cell culture instead of autoclaving. A microfluidic culture based on spontaneous vasculogenesis of endothelial cells was used to create MVN. A dense HUVEC cell suspension of 3 × 10^6^ cells/mL of hydrogel composed of fibrin and collagen was injected into the repository of microfluidic devices. Bovine fibrinogen (Sigma Aldrich, Singapore F8630-1G) at 5 mg/mL was used in 1x phosphate-buffered saline (PBS). The seeding media contained sodium hydroxide neutralized collagen I (collagen I from rat tail in acetic acid, BD354236) at 0.2 mg/mL, the non-specific metalloproteinase inhibitor GM6001 (Millipore CC1010) at a concentration of 0.15 units (U)/mL, and bovine thrombin (Sigma Aldrich T4648-1KU) at a concentration of 0.5 U/mL. Collagen I is helpful to engineer the hydrogel with extracellular matrix components [19]. Collagen I induces motility in endothelial cells for network formation [20]. Thrombin stock solution of 2 mg/mL was prepared of which 4 μL were supplemented to obtain 996 μL of media with a final concentration of roughly 0.5 U/mL. Care must be taken to choose a hydrogel composition that is conducive for cell survival [21] and vasculogenesis while methods exist to engineer the porosity and mechanical properties of hydrogels [22,23]. The seeding media containing 6 × 10^6^ cells/mL was mixed with fibrinogen in a ratio of 1 to 1 in small batches immediately prior to filling the gel regions of the devices. The two media channels were seeded with an endothelial cell suspension of 6 × 10^6^ cells/mL in EGM-2 cell culture media after solidification of the central gel region. Devices were perfused with EGM-2 media (Lonza, Singapore) for maintenance every 24 h.

### 2.2. General Cell Culture and Cell Expansion

HUVECs (pooled donor, Lonza, Singapore) were cultured in EGM-2 (Lonza, Singapore). Cells were generally expanded to p+4 prior experimentations, which is an early passage with a good capacity to undergo spontaneous vasculogenesis or angiogenic sprouting [24] in our hands. Human mammary adenocarcinoma cells MDA-MB-231 (American Type Culture Collection, ATCC) were cultured in Dubelcco Modified Eagle Medium (DMEM) supplemented with 10% FBS and 1% Pen-Strep (all from Life Technologies, Singapore).

### 2.3. Cancer Cell Extravasation Assay

The assay was performed according to previously established protocols [8]. In brief, human mammary adenocarcinoma cells (MDA-MB-231) were trypsinized and suspended in EGM-2 cell culture media at a final concentration of 4 × 10^5^ cells/mL. This cell suspension was seeded into microvascular networks grown through spontaneous vasculogenesis from 3-dimensional HUVEC suspension in the absence of any supporting cells. Microvascular networks were used on the 6th day after HUVEC seeding. Forty μL of cancer cell suspension was placed into the media channel at one side of the microfluidic device in such a way that it created a slight hydrostatic pressure difference across the central gel region. This hydrostatic pressure difference drove cancer cells into the microvascular networks through the openings to the media channel. The microfluidic cultures were placed into a cell culture incubator immediately after cancer cell seeding and were allowed to equilibrate for 45 min. A certain fraction of MDA-MB-231 cells were mechanically entrapped or otherwise bound to the endothelial interface. Non-adherent cells were washed off by applying cell culture media to the channel opposite to that in which cancer cells were seeded, twice. Microvascular networks with entrapped MDA-MB-231 cells were cultured with EGM-2 media and kept on an incubator stage at 37 °C and 5% CO_2_ for in situ life confocal imaging. Whether life-imaging of lectin-stained MVN within microfluidic devices is possible needs to be evaluated in the future. Biological effects potentially induced through lectin binding will need to be understood in life-cultures prior to utilization for cancer cell extravasation studies. Here, microvascular networks with entrapped MDA-MB-231 were cultured in stationary incubators and fixed after 12 h with 4% paraformaldehyde solution in 1× PBS prior to lectin staining.

### 2.4. Immunocytochemistry and Phalloidine Staining

Microfluidic cultures were fixed with 4% paraformaldehyde solution washed with buffer and incubated in 3% BSA blocking solution in PBS for 60 min. This was followed by primary antibody incubation at respective dilution in PBS for 1.5 h at room temperature. After washing with PBS, secondary antibodies were applied for 30 min at their respective dilution containing 4′,6-diamidino-2-phenylindole (DAPI)for nuclei staining (DAPI channel not shown in confocal images). The washing steps were conducted with the aid of 2 cm hydrostatic pressure to create flow across the gel regions to accelerate the removal of non-bound antibodies. Phalloidine (AlexaFluor 488 phalloidin A12379 and AlexaFluor 594 A12381; Molecular Probes, Life Technologies, Singapore) staining required additional permeabilization with cold methanol after paraformaldehyde fixation. Paraformaldehyde fixation needs to be performed first to prevent the collapse of fibrin and collagen composite hydrogels upon exposure to methanol. Lipid staining was used as a fast and convenient tool to stain HUVECs (red) and MDA-MB-231 cells (green) prior to seeding. Life cell staining kits (Sigma Aldrich, Singapore, PKH67/PKH26) were used according to the manufacturer’s instructions. Lectin staining was performed similarly to immunocytochemistry except that blocking was performed with 5% BSA in PBS for 8 h prior to staining with 2 mg/mL lectin tetramethylrhodamine isothiocyanate (TRITC) conjugate (*Bandeiraea Simplicifolia*, L5264 Sigma Aldrich, Singapore) in 1× PBS. Heparan Sulfate Proteoglycan (HSPG) primary antibody, monoclonal in mouse (clone A76), was purchased from Abcam (Hong Kong, ab26265) and used in a dilution of 1:100 in PBS. Alexa Fluor^®^ 488 goat anti-mouse IgG (H+L) highly cross-adsorbed and Alexa Fluor^®^ 594 F(ab’)2 Fragment of Goat Anti-Rabbit IgG (H+L) was bought from Life Technologies (Singapore, S34253 and A-11072, respectively) and used in a dilution of 1:400 in PBS.

### 2.5. Confocal Imaging

An Olympus Multiview 1000 system equipped with an incubator stage was used. Laser power, aperture (50 μm to 150 μm), and the step width were reduced to a minimum (0.5 μm to 2 μm), while the resolution was 1024 × 1024 pixels. Images were analyzed with IMARIS software.

## 3. Results and Discussion

**Figure 1:**
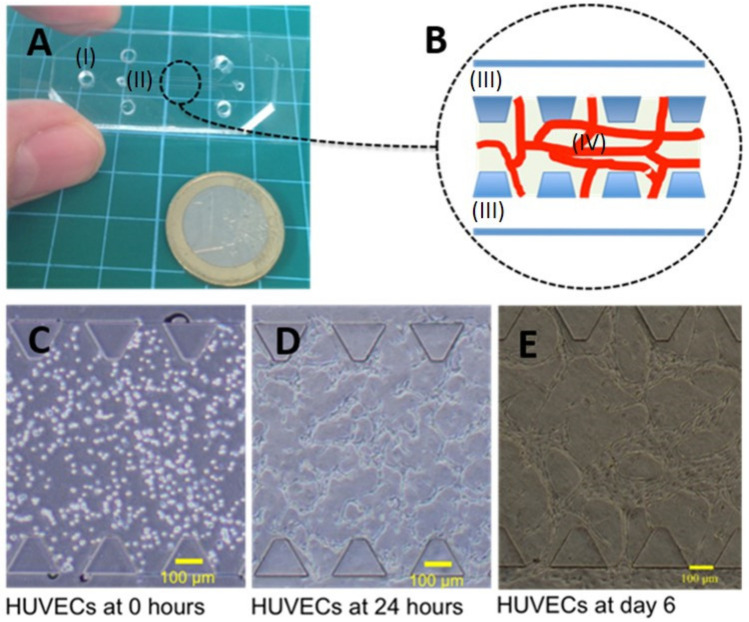
A simple microfluidic chip was used to create microvascular networks (MVNs). The chip is comprised of filling ports for cell culture media (I) and for filling the hydrogel precursor solution with human umbilical vein endothelial cells (HUVEC) suspension (II) as shown in panel (**A**). The chip is comprised of adjacent channels for cell culture media (III) and a central region for confining the hydrogel hosting cellular structures (IV) as shown in panel (**B**). The central gel region confines a fibrinogen-based and collagen I supplemented hydrogel suitable to support microvascular networks created through spontaneous vasculogensis. The temporal progression of HUVEC cultures during spontaneous vasculogenesis is shown. 3D suspension of HUVECs within the hydrogel (**C**), migratory re-arrangement after 24 h (**D**) followed by lumen formation and partial retraction (**E**) can be observed within 6 days of culture.

Seeding of HUVECs into a hydrogel with suitable mechanical properties [25] that is confined within a microfluidic device Figure 1A,B, leads to spontaneous vasculogenesis (Figure 1C–E). In absence of any supporting cells that help to stabilize the network, MVNs exhibit pruning and retract into a few structures with a large diameter, occasionally exceeding 100 µm as shown in Figure 1E. The same figure panel shows that despite network retraction some openings of endothelial lumens to the media channel are preserved. This generally allows perfusion with cancer cells for extravasation studies. Previous works showed that co-culture with fibroblasts, mesenchymal stem cells, or mural cells was necessary to stabilize microvascular networks. Stabilization in these previous studies was mediated through specific matrix proteins or soluble factors [26]. This resulted in fewer vessels with larger lumens in in vitro cultures [27]. Previous studies typically seeded hMSCs into direct contact to stabilize MVN. Alternatively, spatially separated microfluidic co-culture devices were used to stabilize microvascular networks through paracrine signaling of e.g., human dermal fibroblast cells.

However, glycocalyx shedding is known to easily occur upon external stimulus such as paracrine signaling. One example is the paracrine secretion of Angiopoietin 2 (Ang-2) [28]. Ang-2 is known to be secreted by some fibroblasts [29] and human mesenchymal stem cells [30]. The effect of co-cultures of these cells with the glycocalyx MVN in microfluidic cultures seems not to have been investigated so far. While the endothelial glycocalyx of tissue explants appears to be fully functional in the presence of these cells, this may not necessarily be the case for microfluidic cultures that are not subjected to the same physiological balance. Most in vitro works on endothelial glycocalyx utilize pure 2-dimensional endothelial cell cultures. Based on these considerations, a simple experimental set-up solely comprising of HUVECs and MDA-MB-231 breast cancer cells were chosen to address the role of the endothelial glycocalyx in cancer cell extravasation in the present study. The simplicity of this design and limiting potential effects on the endothelial glycocalyx was preferred over network stability and other functional parameters such as the permeability [15] of the microvascular networks.

**Figure 2:**
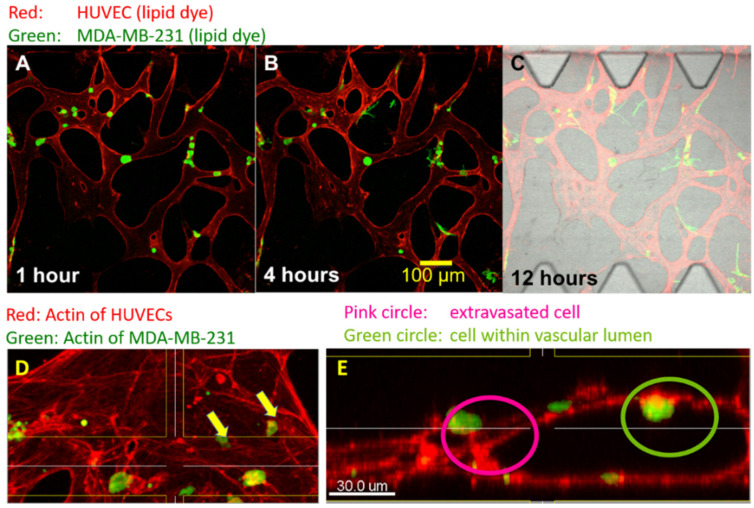
Essential steps in microfluidic-based cancer cell extravasation assays are shown. The cell membrane of HUVECs is stained red with a lipid dye (**A**–**C**) and actin filaments are stained with AlexaFluor 594 labeled phalloidin (**D**,**E**). MDA-MB-231 cancer cells are labeled green. MVNs composed of HUVECs in hydrogels form openings to the media channels. This allows perfusion with MDA-MB-231 cancer cells. The cancer cells then become entrapped within the microvascular lumens (**A**). The entrapped cancer cells protrude filopodia through the endothelial barrier into the perivascular space within 4 h (**B**). Cancer cells are rarely observed to start extravasation later than 12 h after arrest within endothelial structures, and extravasated cells have a stretched morphology compared to round non-extravasated cells (**C**). Optical sectioning (**D**) with confocal laser scanning microscopy is used to clearly establish the extravasation status of extravasated and non-extravasated cancer cells (**E**). Actin staining (red) of endothelial cells was used in figure panels D and E.

A typical cancer cell extravasation assay utilizing solely HUVEC based MVN is shown in Figure 2. The extravasation status can be clearly established by a change in cancer cell morphology from round circular shape to elongated shape upon extravasation (Figure 2A–C), and by confocal sectioning, indicating the presence within or outside the vascular lumen (Figure 2D,E).

**Figure 3:**
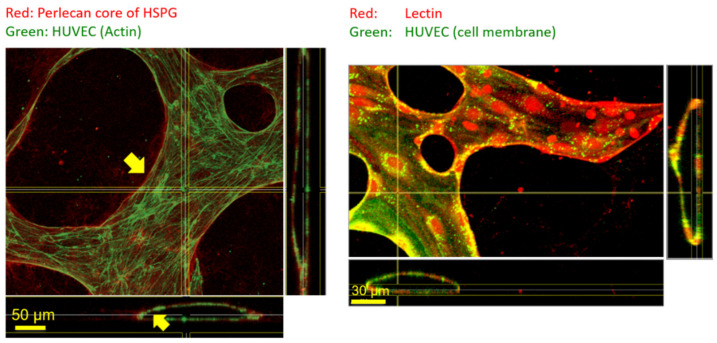
The difference between the immunostained core protein of perlecan, a basement membrane-based heparan sulfate proteoglycans (HPSG) on the left panel, and lectin stain on the right panel is shown. Immunostaining of core proteins of perlecan primarily revealed structures on the abluminal side away from the cyto-skeleton. In contrast, lectin staining co-localizes with that of the cell membrane with much lesser staining of structures within the hydrogel on the abluminal side.

The carbohydrates of the endothelial glycocalyx are anchored to endothelial cells through core proteins such as syndecan and glypican [1]. To understand the specificity of the lectin *Bandeiraea Simplicifolia* to endothelial glycocalyx, we were interested to learn about the potential presence of glycosaminoglycans within the perivascular space. Glycosaminoglycans such as heparan sulfate are known to be bound to perlecan within the basement membrane. Our experimentation has shown that immunostaining works well for perlecan, which is observed primarily on the abluminal side within the perivascular space of microfluidic MVN cultures. Lectin from *Bandeiraea Simplicifolia* appears to strongly co-localize with lipid stain of endothelial cell culture and does not stain structures in the perivascular space to the same extent (Figure 3) in our studies. This indicates a certain specificity of *Bandeiraea Simplicifolia* towards the endothelial glycocalyx of MVN within microfluidic cultures.

**Figure 4:**
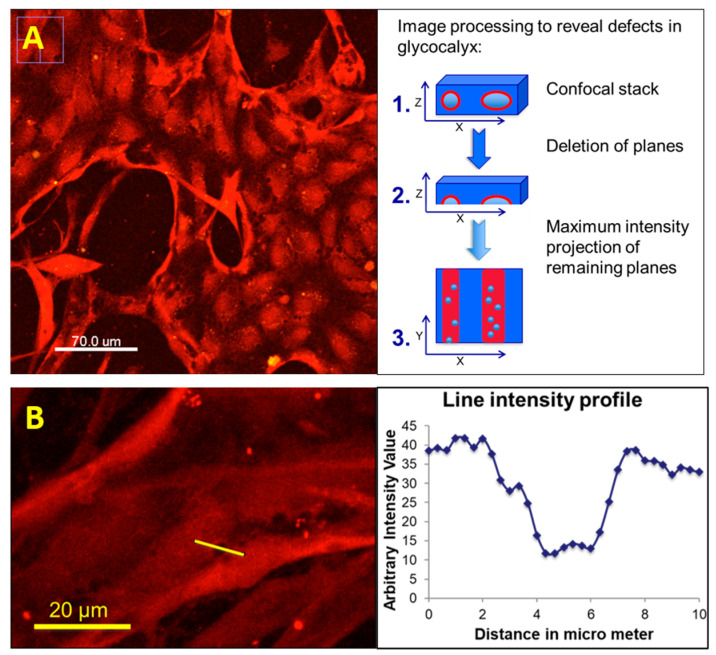
Here, lectin-TRITC stained (*Bandeiraea Simplicifolia*) are depicted. The procedure for the treatment of confocal stacks to reveal glycocalyx defects is described. The selection of confocal planes that represent approximately one-half of the microvessels in a region of interest (ROI) and performing a maximum intensity projection of these confocal planes (**A**) is shown. Noteworthy, the total intensity projection of confocal stacks reveals the size of observed glycocalyx defects to be several microns (**B**).

Our data analysis revealed that lectin staining enabled the visualization of glycocalyx defects (Figure 4). These glycocalyx defects became visible through performing a maximum intensity projection over a certain number of confocal stacks. The number was selected so that one-half of the vascular structure in a region of interest (ROI) was captured. Performing a maximum intensity projection over the full luminous structure (top and bottom) made it difficult to recognize these glycocalyx defects in our data sets. Plotting a line intensity profile indicated the approximate size of the glycocalyx defects to be about 1 to 5 µm in diameter. Our observations also indicated that these glycocalyx defects were in the vicinity of cancer cells. This certainly would be an interesting starting point for more quantitative investigations that could be facilitated by studying glycocalyx defects in 2-dimensional endothelial cell cultures using lectin staining in the presence and absence of cancer cells. A very recent work by one of us [2] describes that cancer cells shed their glycocalyx, which then absorbs at the endothelial wall that presumably must have shed its own glycocalyx to provide grounds for the extravasation event. Whether these two observations correlate still needs to be investigated.

**Figure 5:**
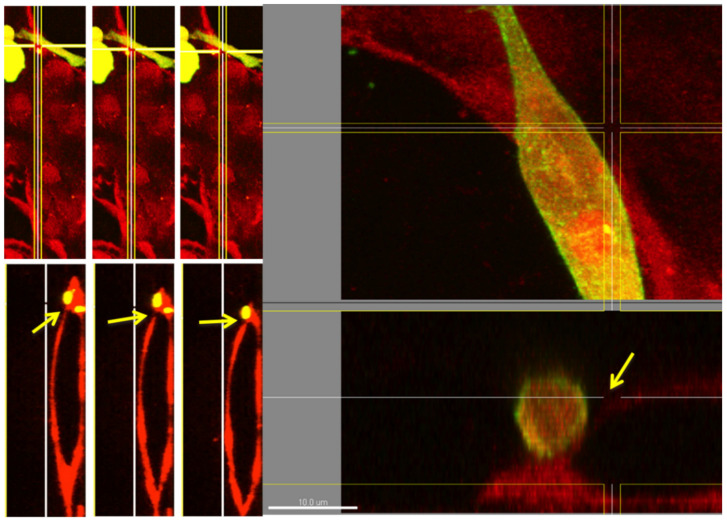
An extravasated cell (elongated shape, approximately 10 µm in diameter at maximum) outside an endothelial lumen at 200× magnification (left images) and at 600× magnification (right images) is shown. The cell membrane of MDA-MB-231 cancer cells was stained with a green, fluorescent lipid stain prior to seeding. The weakened lectin (*Bandeiraea Simplicifolia*) stain of endothelial lumens at the side of extravasation is clearly visible (the areas pointed out by arrows).

**Figure 6:**
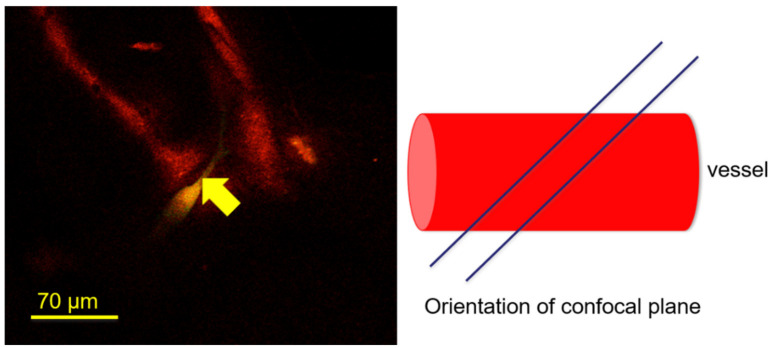
A single confocal plane capturing the lectin (*Bandeiraea Simolicifolia*) stained endothelial glycocalyx (red), as well as a lipid stained (green) cancer cell towards the end of the extravasation process, is shown here. The weakened lectin stain (yellow arrow) could be visualized within a single confocal plane.

High-resolution imaging of glycocalyx stained microvascular networks indicates a weakened lectin stain at the side of cancer cell extravasation (Figure 5). Variation in positioning the confocal sectioning tool of the imaging software indicated a sharply contrasted glycocalyx defect of only a few microns in size. This glycocalyx defect occurred close to the extravasation side in an otherwise strongly stained specimen. This weakened stain or glycocalyx defect is apparent even in single confocal slices (Figure 6).

## 4. Conclusions

Previous works by others used lectin stains primarily for semi-quantitative assessment of endothelial glycocalyx shedding in the presence of cancer cells on a large area (2-dimensional cultures) [3] or within singly confocal planes (3D microfluidic culture) [31]. In contrast, our present work shows that lectin staining can be used for high-resolution imaging, showing detailed spatial features of the glycocalyx at the site of cancer cell extravasation, which is characterized by a weakened lectin stain. This provides an indication that lectin staining of the endothelial glycocalyx may be suitable to elucidate the spatiotemporal role of the endothelial glycocalyx during cancer cell extravasation with high-resolution confocal microscopy. Careful experimental design would need to be applied to such studies taking unspecific shedding of the endothelial glycocalyx into account. In addition, the dynamic complexity of endothelial glycocalyx in presence of cancer cells needs to be considered [32]. Whether lectin binding induces a biological response in life culture also needs to be evaluated prior to life-imaging of cancer cell extravasation. The lectin from *Bandeiraea Simplicifolia* used in this study has a secondary affinity for N-acetyl-α-D-glactosaminyl moieties and a strong affinity for α-D-galactosyl residues, which makes it a preferential stain for microvascular endothelial glycocalyx [33] and may allow capturing e.g., neuraminidase mediated glycocalyx defects. This appears plausible since the *Bandeiraea Simplicifolia* lectin is known to reduce binding to endothelial glycocalyx upon neuraminidase treatment [34]. However, any correlation of the observed glycocalyx defects in the presence of cancer cells with any specific enzyme can presently only be hypothesized and needs future experimental verification. Lectins are a diverse and very established class of stains for tissue explants and their sections. However, lectins appear not to have been used much as a stain in modern tissue culture techniques such as in 3D cell cultures in microfluidic devices. Although the presented data are of preliminary nature, we have shown the suitability of high-resolution imaging using lectin stains to reveal the spatiotemporal role of the carbohydrate interface in complex biological processes such as cancer cell extravasation.

The careful use of lectins prospects opportunities to address key events in cancer cell extravasation at the carbohydrate interface using differential lectin staining that has different affinities and specificities.

## Data Availability

The data presented in this study are available on request from the corresponding author.
